# All-angle reflectionless negative refraction with ideal photonic Weyl metamaterials

**DOI:** 10.1038/s41377-022-00972-9

**Published:** 2022-09-19

**Authors:** Yachao Liu, Guo Ping Wang, John B. Pendry, Shuang Zhang

**Affiliations:** 1 College of Electronics and Information Engineering, Shenzhen University, 518060 Shenzhen, China; 2 School of Physics & Astronomy, University of Birmingham, Birmingham, B15 2TT UK; 3 The Blackett Laboratory, Department of Physics, Imperial College London, London, SW7 2AZ UK; 4 Department of Physics, University of Hong Kong, Hong Kong, China; 5 Department of Electrical & Electronic Engineering, University of Hong Kong, Hong Kong, China

**Keywords:** Metamaterials, Nanophotonics and plasmonics

## Abstract

Negative refraction, an unnatural optical phenomenon in which the incident and the refracted waves reside on the same side of the surface normal, has been demonstrated with the invention of negative index media based on artificially engineered photonic structures called metamaterials. It has received wide attention due to its potential applications in imaging, nonlinear optics, and electromagnetic cloaking. However, it is highly challenging to realize negative refraction operating at all angles and with the perfect transmission. In this work, leveraging the recent development in topological photonics, we propose to realize reflectionless negative refraction for all incident angles with a topological metamaterial. The proposed metamaterial possesses two Weyl points of opposite topological charges. By interfacing the metamaterial with a perfect electric conductor (PEC) or a perfect magnetic conductor (PMC), the Fermi arc connecting the two Weyl points can take the form of a half-circle possessing a positive or a negative refractive index. Importantly, due to the topological protection, there is no reflection at the interface between the PEC and PMC covered areas, leading to the observation of all-angle negative refraction without reflection at the boundary. Our work provides a new platform for manipulating the propagation of surface waves, which may find applications in the construction of integrated photonic devices.

## Abstract (web summary)

A robust negative index flat lens which collects all energy launched from the source is constructed with the ideal photonic Weyl metamaterials.

## Graphical abstract

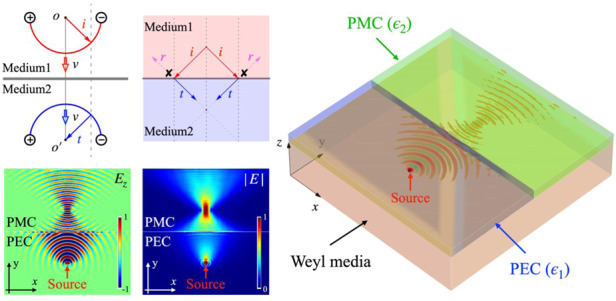

## Introduction

Negative refraction is a highly counter-intuitive electromagnetic effect that had been long believed to be impossible^1^. The rapid development of metamaterials in recent years has led to its demonstration in various photonic systems, such as the double-negative metamaterials^2–7^, photonic crystals^8–11^, chiral metamaterials^12–14^, surface plasmons^15–18^, and hyperbolic materials^19–23^. This novel effect has also been extended to other physical systems, such as acoustic and electronic systems^24–28^. However, most previous demonstrations of negative refraction have suffered from low transmission due to impedance mismatch and limited range of incident angle, whereas a combination of all-angle and perfect transmission will greatly facilitate the application of this novel effect.

Recently, topological principles have been implemented in photonics via the engineering of novel artificial photonic structures^29,30^, such as the Weyl and Dirac metamaterials^31–34^ and photonic topological insulators^35^. These new photonic systems host exotic surface states with topology-protected propagation characteristics^32–35^. It was reported in a seminal work by He et al. that negative refraction of the surface waves without any reflection could occur across certain hinges in phononic Weyl systems^36^. However, this dispersion of the surface modes is highly anisotropic, with negative refraction only occurring for a very narrow incident angle, i.e., a negative refractive index cannot be assigned. A physical system for achieving All-angle REflectionless Negative refraction (AREN) has yet to be discovered.

**Fig. 1:**
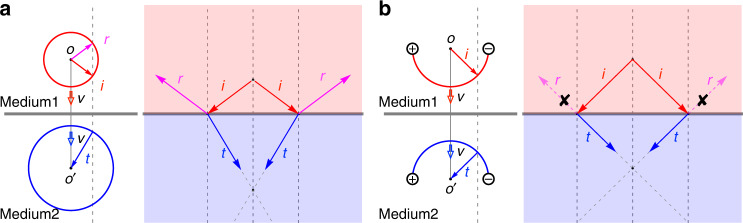
Illustrations of negative refraction of different mechanisms. **a** Negative refraction with conventional negative index medium. The left and right panels show the negative refraction in the momentum space and in the real space, respectively. Arrows labeled by *i*, *t*, and *r* represent the wave vectors of incident, refracted, and reflected waves respectively. The direction of group velocity *v* is indicated by the hollow arrows. **b** All-angle REflectionless Negative refraction (AREN) for surface states at the interface of a minimal ideal Weyl system. EFCs in the configuration of open semicircles are illustrated, where the reflected wavevector (*r*) is forbidden because the reflection state does not exist. Therefore, all energy launched from the source is collected to form the image

Here, we propose to realize AREN in an ideal photonic Weyl system that contains only two ideal Weyl points (WPs) located at the same frequency. The principle of AREN, together with that of conventional negative refraction with a negative refractive index medium for comparison, is illustrated in Fig. 1. The refraction and reflection are governed by the momentum conservation in the in-plane direction (illustrated by the dashed gray lines), and the directions of their respective group velocities. In the conventional negative refraction, reflection is generally allowed, and its amplitude is determined by the impedance mismatch between the incident and the transmitting media (Fig. 1a). To realize AREN, we consider the equifrequency contour (EFC) of a Fermi arc, which is an open arc connecting between the projections of a pair of WPs with opposite topological charges in the surface Brillouin zone (BZ). With appropriate boundary conditions at the surface provided by the carefully chosen surrounding medium, the Fermi arcs can form a semicircle. Across the boundary, the Fermi arc can be controlled to occupy only the top half or the lower half of the circle, with the group velocity pointing outward or inward, respectively, i.e., the sign of the effective refractive index is flipped (Fig. 1b). As such, there would be no reflection state across the boundary, and negative refraction of perfect transmission can be achieved. In contrast to conventional all-angle negative refraction, our work opens the door to a robust negative index flat lens which collects all energy launched from the source.

## Results

### Effective Hamiltonian

**Fig. 2:**
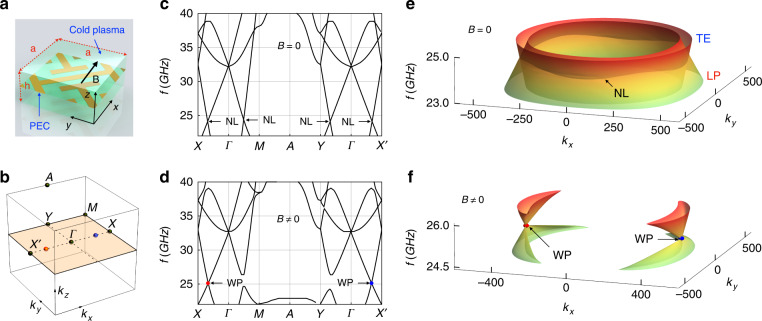
Realization of the minimal ideal photonic Weyl metamaterial. **a** Unit cell with in-plane period *a* = 5 mm and thickness *h* = 2 mm. The lengths of the long and short arms of the metallic (PEC) structure are 1.1*a* and 0.5*a*, respectively. The width of all metallic (PEC) wires is 0.1*a*, and the thickness is 35 μm. The metallic (PEC) structure is embedded in a magnetized plasma with electron density 2 × 10^12^ cm^−1^ under a 0.5 *T* static magnetic field *B* in the *x* direction. **b** Bulk BZ with the high-symmetry points [involved in **c** and **d**] highlighted by black dots. The two WPs [red and blue dots, observed in **d**] are located along the Γ−*X* and Γ−*X*′ directions. **c** Simulated band structure along high-symmetry lines when the TRS is respected (*B* = 0). Three degenerated points are found at the frequency *f*_*n*_ = 24.1 GHz, reminding us of the NL in the *k*_*x*_–*k*_*y*_ plane. **d** Same as **c** but for the broken TRS (*B* ≠ 0). Two WPs (red and blue dots) are observed at the frequency *f*_*w*_ = 25.1 GHz. **e**, **f** 3D views of the band structures in the *k*_*x*_–*k*_*y*_ plane for the NL and Weyl metamaterials, respectively, wherein the NL and WPs are visualized

A system consisting of only two Weyl points requires the breaking of time-reversal symmetry (TRS)^37,38^. Here the ideal Weyl metamaterial is constructed by embedding a periodic metallic structure with non-symmorphic symmetry into magnetized cold plasma, wherein the TRS is achieved via gyro-electric response of plasma under an external DC magnetic field (*B*)^39,40^, as shown in Fig. 2a. The unit cell consists of two I-shape metallic cut-wire resonators along orthogonal directions with a dimension of 5 × 5 × 2 mm^3^. Because of the dispersion (*ω*-dependence) of the permittivity tensor of the magnetized plasma (see Materials and Methods), general eigenvalue solvers (COMSOL and CST Microwave Studio) cannot be applied to this metamaterial. To show the photonic band structure, a complex k-band method is developed to circumvent the ω-dependent eigenvalue problem in our simulations^41,42^ [see Materials and Methods, and Sec. I in the supplementary information (SI) for details].

In the absence of the DC magnetized field (*B* *=* 0), the band structure of the system along high-symmetry lines is shown in Fig. 2c, where the positions of related high-symmetry points are shown in Fig. 2b. This band structure reveals that degeneracies are present in the *k*_*x*_–*k*_*y*_ plane. The band structure in the *k*_*x*_–*k*_*y*_ plane shows the presence of a circular nodal line (NL) (Fig. 2e), which is formed by the crossing between the longitudinal bulk plasma (LP) mode and the transverse electric (TE) mode (see SI, Fig. S1 for field plots of the TE and LP modes). By introducing a glide symmetry into the cut-wire metamaterial design, a negative dispersion for the LP mode is achieved, leading to NL of type-I^43^. Using TE mode and LP mode as the basis, the effective Hamiltonian of this NL system can be expressed as:

1 $$H = w\left( {k_r - k_{NL}} \right)\sigma _0 + v_1\left( {k_r - k_{NL}} \right)\sigma _z + v_2k_z\sigma _x$$

where *v*_1,2_ are the group velocities, σ_*x,y,z*_ are the Pauli matrices, σ_0_, is the 2-by-2 identity, *w* is a factor that accounts for the tilt of the crossing bands, $k_r = \sqrt {k_x^2 + k_y^2}$ is the wavevector along the radial direction in the *k*_*x*_–*k*_*y*_ plane, *K*_*NL*_, is the radius of the circular NL. Eigenstates of the system can be generally expressed as $c_1\left| {TE}\rangle \right. + c_2\left| {LP}\rangle \right.$, where *c*_1_ and *c*_2_ are real functions of ***k***. In the *k*_*x*_–*k*_*y*_ plane (i.e., *k*_*z*_ = 0), the eigenmodes are purely TE (*c*_2_ = 0) and LP (*c*_1_ = 0) modes, where the TE mode and LP mode form the linear crossings [see SI, Sec. V for the derivation of the effective NL Hamiltonian, and Figs. S2 and S3 for the corresponding 3D band structures and EFCs].

When a static magnetic field is applied in the x direction (*B* ≠ 0), the permittivity of the magnetized plasma takes the form of a gyro-electric tensor, which breaks the TRS in our design. The solved band structure along high-symmetric lines is presented in Fig. 2d, which shows the presence of two band degeneracies (the WPs) marked by colored points, red and blue for the opposite topological charges, respectively. These two WPs reside at the same frequency ($f_w = 25.1\;{\mathrm{GHz}}$) and are relatively far away from the other bands, thus forming an ideal minimal Weyl system. The linear crossing of the WPs is also confirmed by the plots of the three-dimensional (3D) band structure in the *k*_*x*_–*k*_*y*_ plane, as shown in Fig. 2f. Due to the breaking of TRS in the presence of an external magnetic field, the NL is gapped everywhere except for the location of WPs along the direction of the magnetic field (*k*_*x*_ direction).

The effective Hamiltonian of the magnetized system around the Weyl frequency can be written as,

2 $$H = w(k_r - k_{NL})\sigma _0 + v_1(k_r - k_{NL})\sigma _z + v_2k_z\sigma _x + m(\theta )\sigma _y$$

where *m*(*θ*) is a *θ*-dependent effective mass that opens up bandgap everywhere except for *θ* = 0 and *π*, *m*(*θ*) = 0, corresponding to the locations of the two Weyl points. The bandgap is largest for *θ* = *π*/2 and *θ* = 3*π*/2, i.e., along the $\pm k_y$ directions, where the effective mass reaches its maximum *m*_0_. Hence, the effective mass can be approximated as a sinusoid function *m*(*θ*) = *m*_0_*sinθ*, which is also confirmed numerically [see SI, Fig. S4 for details]. The opened bandgap arises from the coupling between the TE mode and LP mode, leading to hybridized modes with a transverse spin oriented along the azimuthal direction^44^. Thus, in the *k*_*y*_–*k*_*z*_ plane, there are two valleys located at positive and negative *k*_*y*_, respectively, [see SI, Sec. IV for the derivation of the effective Weyl Hamiltonian, and Figs. S2 and S3 for the corresponding 3D band structures and EFCs].

### Semicircle Fermi arcs

Next, we derive the dispersion of the surface states under perfect electric conductor (PEC), and the perfect magnetic conductor (PMC) boundary conditions based on the effective Hamiltonian formulated above. The effective Hamiltonian of the ideal Weyl system is also expressed as

3 $${{{\mathrm{H}}}} = \left[ {\begin{array}{*{20}{c}} {v_1(k_r - k_{NL})} & {v_2k_z - i\,m_0{\mathrm{sin}}\theta } \\ {v_2k_z + i\,m_0{\mathrm{sin}}\theta } & { - v_1(k_r - k_{NL})} \end{array}} \right]$$

Tilting of the crossing bands can be neglected, i.e., *w* = 0, without affecting the main results. Here, without losing generality, we set *v*_1_ = *v*_2_ = 1, and the eigen-equation becomes

4 $$\left[ {\begin{array}{*{20}{c}} {k_r - k_{NL}} & {k_z - i\,m_0{\mathrm{sin}}\theta } \\ {k_z + i\,m_0{\mathrm{sin}}\theta } & {k_{NL} - k_r} \end{array}} \right]\left( {\begin{array}{*{20}{c}} {c_1} \\ {c_2} \end{array}} \right) = {\rm E}\left( {\begin{array}{*{20}{c}} {c_1} \\ {c_2} \end{array}} \right)$$

where E is the eigenvalue. This equation can be further expanded as,

5 $$\left( {k_r - k_{NL} - E} \right)c_1 + \left( {k_z - i\,m_0{\mathrm{sin}}\theta } \right)c_2 = 0$$

6 $$\left( {k_z + i\,m_0{\mathrm{sin}}\theta } \right)c_1 + \left( {k_{NL} - k_r - E} \right)c_2 = 0$$

**Fig. 3:**
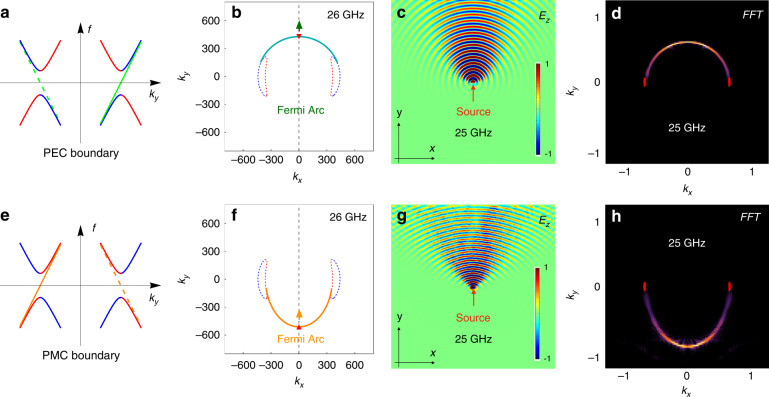
Semicircular surface Fermi arcs in the minimal ideal Weyl system. **a** Schematic dispersion of surface states obtained at the PEC boundary of the Weyl material. Solid and dashed green dispersion lines represent the evanescent (physical) and growing (unphysical) surface states, respectively. The bulk TE mode and LP mode are marked in blue and red, respectively. **b** Expected surface Fermi arc (green curve) under the PEC boundary condition. Red and blue dotted lines show the projected EFCs of bulk states at the same frequency (26 GHz). Colored arrow shows the direction of group velocity of the representative mode (red triangle). **c** Distribution of electric field *Re*(*E*_*Z*_) of the surface wave at the interface between a PEC and the Weyl metamaterial, excited by a point source at 25 GHz. **d** Simulated semicircular EFC of surface states (Fermi arc) in the momentum space obtained by Fourier transformation (*k*_*x*_ and *k*_*y*_ are scaled with $k_0 = \frac{{2\pi }}{{5 \times 10^{ - 3}}}m^{ - 1}$). **e**–**h** Same as **a**–**d** but for the PMC boundary. The evanescent (physical) and growing (unphysical) surface stat**e**s are plotted in solid and dashed orange lines in **e**, respectively

In general, the surface mode is a combination of the TE mode and LP mode (i.e., $c_{1,2} \ne 0$). However, for PEC boundary (i.e., ***n*** × ***E*** = 0, ***n*** is the unit out-normal vector of the boundary wall), the LP mode contains tangential electric field component, and therefore it should be excluded for the construction of the surface mode, i.e., *c*_2_ = 0. As a result, Eqs. (5) and (6) can be solved as $k_z = - im_0{\mathrm{sin}}\theta$ and $E = k_r - k_{NL}$. For 0 ≤ *θ* ≤ *π*, this solution describes an evanescent wave into the Weyl material (along −*z* direction) with a positive group velocity, which is a physical solution for the surface state. While for *π* ≤ *θ* ≤ 2*π*, an unphysical solution describing a growing wave into the Weyl material with a positive group velocity is obtained. For illustration, linear dispersions across the opposite valleys (*θ* = *π*/2 and 3*π*/2) are demonstrated in Fig. 3a to show the physical (solid) and unphysical (dashed) surface states. Thus, the surface Fermi arc at different energy (frequency) can be solved as $k_r = E + k_{NL}$ in the 0 ≤ *θ* ≤ *π* region, which is semicircles as exemplified in Fig. 3b.

On the other hand, for a PMC boundary (i.e., ***n*** × ***H*** = 0), one needs to set *c*_1_ = 0, i.e., there should be no TE component in the surface mode, and Eqs. (5) and (6) can be solved as $k_z = im_0{\mathrm{sin}}\theta$ and $E = k_{NL} - k_r$. For 0 ≤ *θ* ≤ *π*, this solution represents an unphysical growing wave with a negative group velocity, while for *π* ≤ *θ* ≤ 2*π*, the solution is an evanescent wave with a negative group velocity. We show the different solutions located at the opposite valleys *θ* = *π*/2 and 3*π*/2 in Fig. 3e, where the solid and dashed orange lines are the physical and unphysical solutions, respectively. The surface Fermi arc $k_r = k_{NL} - E$ can only be found in the *π* < *θ* ≤ 2*π* region, as shown in Fig. 3f.

Thus far, we have shown that the PEC and PMC boundaries can incur the complementary surface modes for the proposed Weyl metamaterial. Also, these surface modes are located at the same locations as bulk TE mode and LP mode of the original NL system, as schematically shown in Fig. 3b, f, respectively. Moreover, the directions of group velocity (small arrows) in these figures illustrate that the PEC and PMC boundaries of the Weyl material can be treated as positive and negative media, respectively.

### Effective medium model

We next simulate wave propagation inside a large-scale metamaterial system. To this end, we build a nonlocal effective medium model for the minimal ideal Weyl metamaterial with the effective permittivity tensor expressed as:

7 $$\overline{\overline {{\it{\epsilon }}_e}} = {\it{\epsilon }}_0\left[ {\begin{array}{*{20}{c}} {{\it{\epsilon }}_c - {\it{\epsilon }}_m({{{\boldsymbol{k}}}})} & 0 & 0 \\ 0 & {{\it{\epsilon }}_c - {\it{\epsilon }}_m({{{\boldsymbol{k}}}}) - {\it{\epsilon }}_s} & { - i\,{\it{\epsilon }}_d} \\ 0 & {i\,{\it{\epsilon }}_d} & {{\it{\epsilon }}_c - {\it{\epsilon }}_s} \end{array}} \right]$$

where ${\it{\epsilon }}_m( {{{\boldsymbol{k}}}} ) = {\it{\epsilon }}_c\omega _q^2/[ {\omega ^2 - \omega _0^2 + \alpha ( {k_x^2 + k_y^2} )} ]$ takes into account the nonlocal optical response of metallic structure^45–47^ (*∈*_*c*_ is the effective relative permittivity, *α* is a constant for scaling the nonlocal effect, *ω*_0_ and *ω*_*q*_ are the effective resonance angular frequency); ${\it{\epsilon }}_s = \omega _p^2/\left( {\omega ^2 - \omega _1^2} \right)$ and ${\it{\epsilon }}_d = \omega _1\omega _p^2/\left[ {\omega \left( {\omega ^2 - \omega _1^2} \right)} \right]$ arise from the gyro-electric response of magnetized plasma (*ω*_*p*_ and *ω*_1_ are the effective plasma and cyclotron frequencies respectively). We highlight the ***k***-dependence of the parameters in this model because the nonlocal effect plays an important role to form the type-I ideal WP in our system, see SI, Fig. S5 for details of this analysis. A good agreement between the effective medium model and the practical construction is achieved around the Weyl frequency, as shown in detail in the SI (Sec. II and Fig. S6). In the following simulations, the realistic parameters are chosen: ${\it{\epsilon }}_c = 0.83$, $\alpha = 6.8 \times 2\pi \times 10^{15}\,{\mathrm{rad}}^2/s^2$, $\omega _q = 1.8 \times 2\pi \times 10^{10}\,{\mathrm{rad}}/s$, $\omega _0 = 2.22 \times 2\pi \times 10^{10}\,{\mathrm{rad}}/s$, $\omega _p = 5 \times 10^{10}\,{\mathrm{rad}}/s$, and $\omega _1 = 1.22 \times 10^{10}\,{\mathrm{rad}}/s$.

In the simulation, a dipole source is positioned close to the surface of the Weyl metamaterial under different boundary conditions. We numerically obtain the surface electric field component *E*_*z*_ (real part) on the PEC and PMC boundaries (both in the x-y plane), as shown, respectively, in Fig. 3c, g. It is observed that the excited field only propagates upwards in both cases, which is characteristic of the topological properties of the surface states of the Weyl system. The Fourier transformation of the fields provides the distribution of the surface states in the momentum space, which leads to the formation of the Fermi arcs in the shape of semicircles, as shown in Fig. 3d, h. These semicircular Fermi arcs are exactly what we expect, which is also verified by simulations using the real metallic structure, as shown in our SI (Fig. S7). Despite the similarity between the real-space field plots between the PEC and PMC boundary conditions, the locations of the Fermi arcs in these two cases are dramatically different: the Fermi arc of PEC boundary bends upward, while that of PMC boundary bends downward. Consequently, the Fermi arcs under these two different boundary conditions are complementary to each other, i.e., they represent different parts of a full circle. While most metals serve as PEC at microwave frequencies, there are no natural media that can perform the same as PMC. On the other hand, the boundary condition is mainly determined by the impedance of the surrounding medium. A high impedance ($\sqrt {\mu /{\it{\epsilon }}}$) medium can replace the PMC in practical applications^48^, indicating that the PMC boundary can be replaced by a dielectric medium with very small permittivity^49^. This is verified by a simulation using a dielectric medium with ${\it{\epsilon }} = - 0.01$, as shown in the SI (Sec. III and Fig. S8).

### All-angle reflectionless negative refraction

**Fig. 4:**
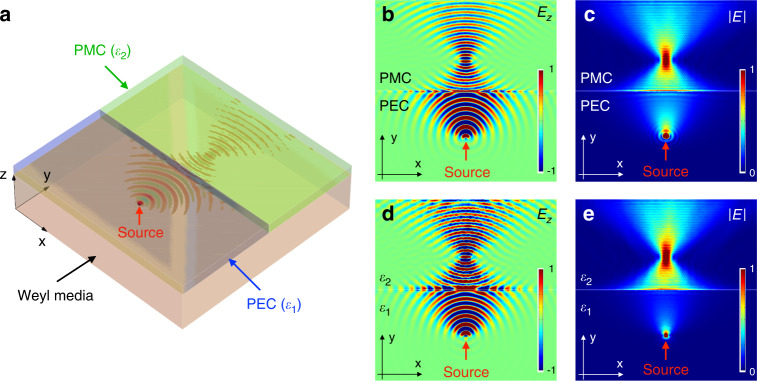
Numerical confirmation of the all-angle reflectionless negative refraction (AREN). **a** Configuration for observing the AREN. Half of the top surface of the Weyl metamaterial is covered by a PEC layer, while the other half is covered by a PMC layer. A point source (electric dipole in *z* direction) is placed at the PEC/metamaterial interface. **b**, **c** Imaging due to the AREN. The distributions of electric field *Re*(*E*_*z*_) and magnitude $\left| {{{\boldsymbol{E}}}} \right|$ at the frequency 25*GHz* are plotted respectively. **d**, **e** The distribution of the electric field and the magnitude obtained by replacing the PEC and PMC layers with two different dielectric layers (∈_1_ = −10 and *∈*_2_ = −0.01)

We finally investigate the refraction of the surface waves at a straight boundary formed between two regions covered by PEC and PMC, respectively, as shown in Fig. 4a. A point source is placed in the region covered by PEC to excite the surface field. The real part of the field and the amplitude distribution are shown in Fig. 4b, c. It is observed that all the surface waves emitted by the pointed source are negatively refracted at the boundary, leading to a sharp focal point in the region covered by the PMC. Meanwhile, there is no reflected wave, demonstrating the topological protection of the negative refraction. Therefore, the AREN predicted in Fig. 1b is observed in our system. By replacing the PMC layers with approximated dielectric layers (${\it{\epsilon }}_2 = - 0.01$), a similar effect is obtained, as shown in Fig. 4d, e.

In order to show the robustness of AREN, the material loss and the equivalent PMC boundary conditions are investigated via simulation. Firstly, by including a material loss in our effective medium model, ${\it{\epsilon }}_c \to {\it{\epsilon }}_c + i\,\gamma$, we examine the robustness of the AREN, as shown in Figs. S9, S11A, and S11B of the SI. By increasing the material loss *γ* from 0 to −0.05, It can be seen that the magnitude of the focused field is reduced while the shape of the focal point is unaffected, illustrating that AREN is not sensitive to the material loss. Secondly, we examine the performance of AREN by changing the value of *∈*_2_, i.e., the permittivity of the dielectric layer for replacing the PMC boundary. The simulated negative refractions are presented in Fig. S10 of the SI. Despite a slight shape deformation of the field distribution when the value of *∈*_2_ deviates from zero, AREN can still be observed for a large range of parameter values (from −0.1 to 0.2). See SI (Fig. S11C, D) for the transverse profiles of the focused fields.

## Discussion

In conclusion, we have realized a minimal ideal Weyl system by introducing the magnetic gyro-electric material (cold plasma) to a nodal-line metamaterial, and shown that the surface modes under PEC and PMC boundary conditions mimic the wave propagation inside a positive and negative index medium, respectively, but with topological protection that can eliminate reflection at the interface. Different from the schemes of all-angle negative refraction in previous studies, impedance-match at the interface between positive and negative media will not change the reflection in our approach. The presented all-angle reflectionless negative refraction can be extended to other frequency regimes by introducing the gyro-electric response with different magnetic media, such as InSb in the terahertz regime^50^. Our work shows that a flat lens that collects all energy from the source is possible, which opens up new opportunities in electromagnetic imaging and beam steering.

## Materials and methods

### Magnetized plasma

The magnetized plasma applied in the metamaterial is simulated by an effective model with gyro-electric response. Expression of the effective relative permittivity tensor can be written as

$$\epsilon = \left( {\begin{array}{*{20}{c}} {s(\omega )} & { - i\,d(\omega )} & 0 \\ {i\,d(\omega )} & {s(\omega )} & 0 \\ 0 & 0 & {p(\omega )} \end{array}} \right)$$

where ${{{\mathrm{s}}}}\left( \omega \right) = 1 - \frac{{\omega _p^2}}{{\omega ^2 - \omega _c^2}}$, $d(\omega ) = \frac{{\omega _c}}{\omega }\frac{{\omega _p^2}}{{\omega ^2 - \omega _c^2}}$, and $p(\omega ) = 1 - \frac{{\omega _p^2}}{{\omega ^2}}$. The plasma frequency is defined as $\omega _p^2 = \frac{{N\,q^2}}{{\epsilon _0m_e}}$, where *N* is electron density, *q* is electron charge, *∈*_0_ is vacuum permittivity, and *m*_*e*_ is electron mass. The cyclotron frequency is defined as $\omega _c = \frac{{q\,B}}{{m_e}}$, where *B* is the steady-state magnetic field. The relative permeability of the magnetized plasma is fixed as *μ* = 1. In our simulations, *N* = 2 × 10^12^ cm^−3^ and *B* = 0.5 *T* are applied, which can both be readily achieved under laboratory conditions.

### Numerical simulations

The complex k-band method used in our simulations can be implemented in the following steps: Firstly, an equivalent field equation is obtained by replacing the field function ***H*** (***r***) with Bloch-wave function ${{{\boldsymbol{h}}}}\left( {{{\boldsymbol{r}}}} \right)\exp \left[ {i\left( {\omega {{{\mathrm{t}}}} - {{{\boldsymbol{k}}}} \cdot {{{\boldsymbol{r}}}}} \right)} \right]$, which makes the wavevector ***k*** become a priori unknown quantity similar to the frequency ω. Then, we write the weak form of the field equation by introducing a test function, which makes sure that the electromagnetic field equation can be solved in software (COMSOL is used in our simulations) based on finite-element method (FEM). In the next step, the real structure of the unit cell (as shown in Fig. 2a) is constructed in the software, periodic boundary conditions are applied for all of the boundaries, and the eigenvalues of ***k*** (magnitude in a selective direction) are solved at fixed frequencies. The field equations used in our simulations can be found in our SI, Sec I.

## Supplementary information

Supplementary Materials

## References

[1] Veselago VG (1967). Electrodynamics of substances with simultaneously negative ε and μ. Usp. Fiz. Nauk.

[2] Shelby RA, Smith DR, Schultz S (2001). Experimental verification of a negative index of refraction. Science.

[3] Smith DR, Pendry JB, Wiltshire MC (2004). Metamaterials and negative refractive index. Science.

[4] Zhang S (2005). Experimental demonstration of near-infrared negative-index metamaterials. Phys. Rev. Lett..

[5] Shalaev VM (2005). Negative index of refraction in optical metamaterials. Opt. Lett..

[6] Shalaev VM (2007). Optical negative-index metamaterials. Nat. Photonics.

[7] Valentine J (2008). Three-dimensional optical metamaterial with a negative refractive index. Nature.

[8] Luo C, Johnson SG, Joannopoulos JD (2002). All-angle negative refraction in a three-dimensionally periodic photonic crystal. Appl. Phys. Lett..

[9] Cubukcu E, Aydin K, Ozbay E, Foteinopoulou S, Soukoulis CM (2003). Negative refraction by photonic crystals. Nature.

[10] Luo C, Johnson SG, Joannopoulos JD, Pendry JB (2003). Negative refraction without negative index in metallic photonic crystals. Opt. Express.

[11] Berrier A (2004). Negative refraction at infrared wavelengths in a two-dimensional photonic crystal. Phys. Rev. Lett..

[12] Pendry JB (2004). A chiral route to negative refraction. Science.

[13] Monzon C, Forester DW (2005). Negative refraction and focusing of circularly polarized waves in optically active media. Phys. Rev. Lett..

[14] Zhang S (2009). Negative refractive index in chiral metamaterials. Phys. Rev. Lett..

[15] Shin H, Fan S (2006). All-angle negative refraction for surface plasmon waves using a metal-dielectric-metal structure. Phys. Rev. Lett..

[16] Lezec HJ, Dionne JA, Atwater HA (2007). Negative refraction at visible frequencies. Science.

[17] Liu Y, Zhang X (2013). Metasurfaces for manipulating surface plasmons. Appl. Phys. Lett..

[18] Lin X (2017). All-angle negative refraction of highly squeezed plasmon and phonon polaritons in graphene–boron nitride heterostructures. Proc. Natl Acad. Sci. USA.

[19] Smith DR, Schurig D, Mock JJ, Kolinko P, Rye P (2004). Partial focusing of radiation by a slab of indefinite media. Appl. Phys. Lett..

[20] Hoffman AJ (2007). Negative refraction in semiconductor metamaterials. Nat. Mater..

[21] Yao J (2008). Optical negative refraction in bulk metamaterials of nanowires. Science.

[22] Poddubny A, Iorsh I, Belov P, Kivshar Y (2013). Hyperbolic metamaterials. Nat. Photonics.

[23] High AA (2015). Visible-frequency hyperbolic metasurface. Nature.

[24] Yang S (2004). Focusing of sound in a 3D phononic crystal. Phys. Rev. Lett..

[25] Zhang S, Yin L, Fang N (2009). Focusing ultrasound with an acoustic metamaterial network. Phys. Rev. Lett..

[26] Zhu R, Liu XN, Hu GK, Sun CT, Huang GL (2014). Negative refraction of elastic waves at the deep-subwavelength scale in a single-phase metamaterial. Nat. Commun..

[27] Lee G-H, Park G-H, Lee H-J (2015). Observation of negative refraction of Dirac fermions in graphene. Nat. Phys..

[28] Chen S (2016). Electron optics with p-n junctions in ballistic graphene. Science.

[29] Lu L, Joannopoulos JD, Soljačić M (2014). Topological photonics. Nat. Photonics.

[30] Ozawa T (2019). Topological photonics. Rev. Mod. Phys..

[31] Lu L (2015). Experimental observation of Weyl points. Science.

[32] Yang B (2018). Ideal Weyl points and helicoid surface states in artificial photonic crystal structures. Science.

[33] Guo Q (2019). Observation of three-dimensional photonic Dirac points and spin-polarized surface arcs. Phys. Rev. Lett..

[34] Yang Y (2020). Ideal unconventional Weyl point in a chiral photonic metamaterial. Phys. Rev. Lett..

[35] Yang Y (2019). Realization of a three-dimensional photonic topological insulator. Nature.

[36] He H (2018). Topological negative refraction of surface acoustic waves in a Weyl phononic crystal. Nature.

[37] Kim M, Jacob Z, Rho J (2020). Recent advances in 2D, 3D and higher-order topological photonics. Light Sci. Appl..

[38] Liu, G.-G. et al. Observation of Weyl point pair annihilation in a gyromagnetic photonic crystal. *arXiv preprint arXiv*:2106.02461, 2021.

[39] Bellan, P. M. *Fundamentals of Plasma Physics.* (Cambridge University Press, Cambridge, 2006).

[40] Gao W (2016). Photonic Weyl degeneracies in magnetized plasma. Nat. Commun..

[41] Davanc M, Urzhumov Y, Shvets G (2007). The complex Bloch bands of a 2D plasmonic crystal displaying isotropic negative refraction. Opt. Express.

[42] Fietz C, Urzhumov Y, Shvets G (2011). Complex k band diagrams of 3D metamaterial/photonic crystals. Opt. Express.

[43] Gao W (2018). Experimental observation of photonic nodal line degeneracies in metacrystals. Nat. Commun..

[44] Peng L (2019). Transverse photon spin of bulk electromagnetic waves in bianisotropic media. Nat. Photonics.

[45] McMahon JM, Gray SK, Schatz GC (2009). Nonlocal optical response of metal nanostructures with arbitrary shape. Phys. Rev. Lett..

[46] Mortensen NA, Raza S, Wubs M, Sondergaard T, Bozhevolnyi SI (2014). A generalized non-local optical response theory for plasmonic nanostructures. Nat. Commun..

[47] Liu Y, Wang GP, Zhang S (2021). A nonlocal effective medium description of topological Weyl metamaterials. Laser Photonics Rev..

[48] Sievenpiper D, Zhang L, Broas RFJ, Alexopolous NG, Yablonovitch E (1999). High-impedance electromagnetic surfaces with a forbidden frequency band. IEEE Trans. Microw. Theory Tech..

[49] Wang T, Luo J, Gao L, Xu P, Lai Y (2014). Equivalent perfect magnetic conductor based on epsilon-near-zero media. Appl. Phys. Lett..

[50] Wang D (2019). Photonic Weyl points due to broken time-reversal symmetry in magnetized semiconductor. Nat. Phys..

