# Supplementary material for: All-angle reflectionless negative refraction with ideal photonic Weyl metamaterials
Source: Light Sci Appl. 2022 Sep 19;11:276. doi: 10.1038/s41377-022-00972-9 (PMC9485223; doi:10.1038/s41377-022-00972-9)
Supplement: Supplementary file 1 — Supplementary Materials [file 41377_2022_972_MOESM1_ESM.docx]

**Supplementary Information for**

**All-angle reflectionless negative refraction with ideal photonic Weyl metamaterials**

Yachao Liu^1, 2^, Guo Ping Wang^1#^, John B Pendry^3†^, and Shuang Zhang^4,5*^

1. *College of Electronics and Information Engineering, Shenzhen University, Shenzhen, 518060, China*
2. *School of Physics & Astronomy, University of Birmingham, Birmingham, B15 2TT, UK*
3. *The Blackett Laboratory, Department of Physics, Imperial College London, London SW7 2AZ, UK*
4. *Department of Physics, University of Hong Kong, Hong Kong, China*
5. *Department of Electrical & Electronic Engineering, University of Hong Kong, Hong Kong, China*

Email: Tel:

Yachao Liu: [yachaoliu@szu.edu.cn](mailto:yachaoliu@szu.edu.cn) +86 13787115809

^#^Guo Ping Wang: [gpwang@szu.edu.cn](mailto:gpwang@szu.edu.cn) +86 13823556131

^†^John B Pendry: [j.pendry@imperial.ac.uk](mailto:j.pendry@imperial.ac.uk) +44 (0)20 7594 7606

^*^Shuang Zhang: [shuzhang@hku.hk](mailto:shuzhang@hku.hk) +852 2859 7944

I. Electromagnetic simulation of the minimal ideal photonic Weyl metamaterials

The wave equation of magnetic field is applied to simulate the electromagnetic waves in our metamaterial,

$\nabla\times\left( \frac{1}{\epsilon}\nabla\times\boldsymbol{H} \right)-\mu\frac{\omega^{2}}{c^{2}}\boldsymbol{H}=0$,

where $\epsilon$ and $\mu$ are the permittivity tensor and permeability tensor respectively, $c=\frac{1}{\sqrt{\epsilon_{0}\mu_{0}}}$ is the speed of light. According to the Bloch’s theorem, the magnetic field can be expressed as:$\boldsymbol{H}\left( \boldsymbol{r} \right)=\boldsymbol{h}\left( \boldsymbol{r} \right)\exp\left[ i\left( \omega t-\boldsymbol{k}\cdot\boldsymbol{r} \right) \right]$. This leads to an equivalent field equation as

$\nabla\times\left( \frac{1}{\epsilon}\nabla\times\boldsymbol{h} \right)-i\nabla\times\left( \frac{1}{\epsilon}\boldsymbol{k}\times\boldsymbol{h} \right)-i \boldsymbol{k}\times\left( \frac{1}{\epsilon}\nabla\times\boldsymbol{h} \right)-\frac{\boldsymbol{k}}{\epsilon}\left( \boldsymbol{k}\cdot\boldsymbol{h} \right)+\frac{k^{2}}{\epsilon}\boldsymbol{h}-\mu\frac{\omega^{2}}{c^{2}}\boldsymbol{h}=0$.

This equation is simplified by introducing a test vector $test(\boldsymbol{h})$ and adding the PEC boundary condition (natural boundary condition). Then we got the weak-form expression that can be implemented by the FEM (finite element method) programs (COMSOL in this work).

Here, the weak-form expression applied in our program is given as

$weak form=test\left( i k_{cx}+c_{cx} \right)e_{11}\left( -i k_{cx}+c_{cx} \right)+test\left( i k_{cx}+c_{cx} \right)e_{12}\left( -i k_{cy}+c_{cy} \right)+test\left( i k_{cx}+c_{cx} \right)e_{13}\left( -i k_{cz}+c_{cz} \right)+test\left( i k_{cy}+c_{cy} \right)e_{21}\left( -i k_{cx}+c_{cx} \right)+test\left( i k_{cy}+c_{cy} \right)e_{22}\left( -i k_{cy}+c_{cy} \right)+test\left( i k_{cy}+c_{cy} \right)e_{23}\left( -i k_{cz}+c_{cz} \right)+test\left( i k_{cz}+c_{cz} \right)e_{31}\left( -i k_{cx}+c_{cx} \right)+test\left( i k_{cz}+c_{cz} \right)e_{32}\left( -i k_{cy}+c_{cy} \right)++test\left( i k_{cz}+c_{cz} \right)e_{33}\left( -i k_{cz}+c_{cz} \right)-\mu k_{0}^{2}\left[ test\left( H_{x} \right)H_{x}+test\left( H_{y} \right)H_{y}+test\left( H_{z} \right)H_{z} \right]$.

where ($h_{x}$, $h_{y}$, $h_{z}$) is the magnetic field $\boldsymbol{h}$; $i$ is the imaginary unit, $e_{ij}$ (i,j=1,2,3) is the components of permittivity tensor; $k_{ci}$ (i=x,y,z) is the i-th component of $\mathbf{k}\times\boldsymbol{h}=(k_{y}h_{z}-k_{z}h_{y},k_{z}h_{x}-k_{x}h_{z},k_{x}h_{y}-k_{y}h_{z})$; $c_{ci}$ (i=x,y,z) is i-th component of $\nabla\times\boldsymbol{h}=(\partial_{y}h_{z}-\partial_{z}h_{y},\partial_{z}h_{x}-\partial_{x}h_{z},\partial_{x}h_{y}-\partial_{y}h_{x})$; $\mu$ is the permeability; and $k_{0}=\omega/c$.

II. Wave equation of the effective Weyl medium

As the effective permittivity provided in the main text, the wave equation of electric field is examined in this work, which can be written as

$\nabla\times\left( \frac{1}{\mu}\nabla\times\boldsymbol{E} \right)-\epsilon\frac{\omega^{2}}{c^{2}}\boldsymbol{E}=0$,

where the analytic form of $\epsilon$ is the Eq.(7) in the main text. The nonlocal effect is considered in our model to fit the electromagnetic response of the practical Weyl metamaterial. This makes the above equation cannot be directly solved. Therefore, we introduce an auxiliary vector $\boldsymbol{J}=\left( J_{1},J_{2} \right)$ to simplify the equation in our calculations, which finally makes the wave equation in the form (weak form) as

$\text{weak form=CE}_{x}\text{CT}_{x}+\text{CE}_{y}\text{CT}_{y}+\text{CE}_{z}\text{CT}_{z}+J_{1}test\left( E_{x} \right)++J_{2}test\left( E_{y} \right)+\epsilon_{0}\mu_{0}\epsilon_{c}\omega^{2}{[E}_{x}test\left( E_{x} \right)+E_{y}test\left( E_{y} \right)+E_{z}test\left( E_{z} \right)]+\frac{\epsilon_{0}\mu_{0}\omega^{3}\omega_{q}^{3}{[E}_{y}test\left( E_{y} \right)+E_{z}test\left( E_{z} \right)]}{\omega^{3}-\omega\omega_{0}^{2}}+\frac{i\epsilon_{0}\mu_{0}\omega^{2}\omega_{0}\omega_{q}^{2}[E_{z}test\left( E_{y} \right)+E_{y}test\left( E_{z} \right)]}{\omega^{3}-\omega\omega_{0}^{2}}+\omega_{1}^{2}[J_{1}test\left( J_{1} \right)+J_{2}test\left( J_{2} \right)]-\alpha[\partial_{x}J_{1}test\left( \partial_{x}J_{1} \right)+ \partial_{y}J_{1}test\left( \partial_{y}J_{1} \right)+\partial_{x}J_{2}test\left( \partial_{x}J_{2} \right)+ \partial_{y}J_{2}test\left( \partial_{y}J_{2} \right)]+\omega^{2}J_{1}test\left( J_{1} \right)+\omega^{2}J_{2}test\left( J_{2} \right)+\epsilon_{0}\mu_{0}\epsilon_{c}\omega^{2}\omega_{o}^{2}[E_{x}test\left( J_{1} \right)+E_{y}test\left( J_{2} \right)]$.

Here, ($E_{x}$, $E_{y}$, $E_{z}$) is the electric field $\boldsymbol{E}$; ($J_{1}$, $J_{2}$) is the auxiliary vector; ($\text{CE}_{x}$, $\text{CE}_{y}$, $\text{CE}_{z}$) is the $\nabla\times\boldsymbol{E}=(\partial_{y}E_{z}-\partial_{z}E_{y},\partial_{z}E_{x}-\partial_{x}E_{z},\partial_{x}E_{y}-\partial_{y}E_{x})$; ($\text{CT}_{x}$, $\text{CT}_{y}$, $\text{CT}_{z}$) is the $test(\nabla\times\boldsymbol{E})$; $i$ is the imaginary unit, $\epsilon_{0}$ and $\mu_{0}$ are the vacuum permittivity and permeability; $\omega$ is the angular frequency; ($\epsilon_{c}, \alpha, \omega_{q}, \omega_{0}, \omega_{p}, \omega_{1}$) are defined in the main text.

Comparison between the responses of the practical Weyl metamaterial and the effective medium is provided Fig. S6, which shows well-agreement between these two different methods.

III. Surface states on the approximated PEC and PMC boundaries

As what we have mentioned in the main text, in the microwave regime, most metals can be treated as PEC. However, the PMC can only be approximated by a dielectric medium with small permittivity. Here, the homogeneous layer with $\epsilon=-10$ and $\epsilon=-0.01$ are used to approximate the PEC and PMC respectively. Figure S8 are the calculated surface electric field and corresponding FFT (fast Fourier transform) results obtained at these boundaries.

IV. Effective Hamiltonian of the Weyl system

As the permittivity tensor given in the main text, we obtained the full Hamiltonian of our Weyl system as

$H=\left[ \begin{matrix} 0 & 0 & 0 & 0 & k_{z} & -k_{y} & \omega_{p} & 0 & 0 & 0 & 0 & 0 \\ 0 & 0 & 0 & -k_{z} & 0 & k_{x} & 0 & \omega_{p} & 0 & 0 & \omega_{o} & 0 \\ 0 & 0 & 0 & k_{y} & -k_{x} & 0 & 0 & 0 & 0 & 0 & -i \omega_{o} & 0 \\ 0 & -k_{z} & k_{y} & 0 & 0 & 0 & 0 & 0 & 0 & 0 & 0 & 0 \\ k_{z} & 0 & {-k}_{x} & 0 & 0 & 0 & 0 & 0 & 0 & 0 & 0 & 0 \\ -k_{y} & k_{x} & 0 & 0 & 0 & 0 & 0 & 0 & 0 & 0 & 0 & 0 \\ \omega_{p} & 0 & 0 & 0 & 0 & 0 & 0 & 0 & \omega_{0} & 0 & 0 & 0 \\ 0 & \omega_{p} & 0 & 0 & 0 & 0 & 0 & 0 & 0 & \omega_{0} & 0 & 0 \\ 0 & 0 & 0 & 0 & 0 & 0 & \omega_{0} & 0 & 0 & 0 & 0 & 0 \\ 0 & 0 & 0 & 0 & 0 & 0 & 0 & \omega_{0} & 0 & 0 & 0 & 0 \\ 0 & \omega_{o} & i \omega_{o} & 0 & 0 & 0 & 0 & 0 & 0 & 0 & 0 & \omega_{1} \\ 0 & 0 & 0 & 0 & 0 & 0 & 0 & 0 & 0 & 0 & \omega_{1} & 0 \end{matrix} \right]$.

Here, for simplicity, $\omega_{1}/\omega\approx1$, $\epsilon_{0}=1$, and $\mu_{0}=1$ are applied in this derivation. The nonlocal parameter is enclosed in $\omega_{0}$, which should be replaced by $\sqrt{\omega_{0}^{2}-\alpha\left( k_{x}^{2}+k_{y}^{2} \right)}$ for the exact form. We note that the basis of the Hamiltonian is ${({\sqrt{\epsilon_{0}\mu_{0}}E}_{x},\sqrt{\epsilon_{0}\mu_{0}}E_{y},\sqrt{\epsilon_{0}\mu_{0}}E_{z},\sqrt{\mu_{0}}H_{x},\sqrt{\mu_{0}}H_{y},\sqrt{\mu_{0}}H_{z},J_{x},J_{y},P_{x},P_{y},J_{1y},P_{1y})}^{T}$, where $J_{x},J_{y},P_{x},P_{y},J_{1y}$, and $P_{1y}$ auxiliary fileds.

Then, we can get the effective Hamiltonian for the Weyl degeneracy. For example, a simple case where $\omega_{p}=1$, $\omega_{0}=\sqrt{3}$, and $\omega_{o}=\omega_{1}=\sqrt{2}$ is considered. Here, we neglected the nonlocality to simplify the expressions. In this case, the position of Weyl point can be derived as $(\pm\sqrt{2(-1+\sqrt{5})},0,0)$, while the two degenerated modes are

$\left| \left. I \right\rangle\right.={[0,\frac{2}{-1+\sqrt{5}},\frac{i(1+\sqrt{5})}{-1+\sqrt{5}},0,\frac{-i}{2}(\sqrt{\frac{10}{-1+\sqrt{5}}}-\sqrt{\frac{4}{-1+\sqrt{5}}}),\sqrt{\frac{2}{-1+\sqrt{5}}},0,\frac{-4}{-1+\sqrt{5}},0,\frac{-2\sqrt{3}}{-1+\sqrt{5}},\sqrt{2},1]}^{T}$;

$\left| \left. J \right\rangle\right.={[\frac{1}{\sqrt{3}},0,0,0,0,0,\frac{1}{\sqrt{3}},0,1,0,0,0]}^{T}$.

The effective Hamiltonian is derived according to the first order expansion, which is

$H_{e}=\delta_{IJ}E_{I}^{0}+\left\langle\left. I \right| \right.\frac{\partial H}{\partial\boldsymbol{k}}d\boldsymbol{k}\left| \left. J \right\rangle\right.$,

where $E_{I}^{0}$ is the eigenvalues of modes $\left| \left. I \right\rangle\right.$ and $\left| \left. J \right\rangle\right.$. Substituting $\left| \left. I \right\rangle\right.$ and $\left| \left. J \right\rangle\right.$ into the first order expansion, we have the effective Hamiltonian as

$H_{e}=\left[ \begin{matrix} 2-\sqrt{110+50\sqrt{5}} p_{x} & \frac{-2 p_{y}+i(1+\sqrt{5})p_{z}}{\sqrt{6(\sqrt{5}-1)}} \\ \frac{-2 p_{y}-i(1+\sqrt{5})p_{z}}{\sqrt{6(\sqrt{5}-1)}} & 2 \end{matrix} \right]$,

which is therefore can be equally written as

$H_{e}=\left( 2-\frac{\sqrt{110+50\sqrt{5}} p_{x}}{2} \right)\sigma_{0}-\frac{\sqrt{110+50\sqrt{5}} p_{x}}{2}\sigma_{x}-2 p_{y}\sigma_{y}+\frac{\left( 1+\sqrt{5} \right)p_{z}}{\sqrt{6\left( \sqrt{5}-1 \right)}}\sigma_{z}$,

where $\sigma_{0}$ is the identity matrix, $\sigma_{x,y,z}$ is the Pauli matrix. The above equation shows that the Weyl point is well defined in our system.

In the last, we derived the effective Hamiltonian for the gaped valley in our system, which is shifted away from the Weyl points. For example, we choose a simplest model where $\omega_{p}=\omega_{0}=\omega_{q}=\omega_{1}=1$, the effective Hamiltonian around the point $(0,1,0)$ is examined, which located in the gap along the $k_{y}$ direction. The lower and upper modes are correspondingly as

$\left| \left. I \right\rangle\right.={[0,\frac{-19}{10},\frac{i5}{2},-2 i,0,0,0,\frac{-37}{10},0,\frac{29}{10},\frac{13}{10},1]}^{T}$;

$\left| \left. J \right\rangle\right.={[\frac{-3}{5},0,0,0,0,1,\frac{3}{5},0,1,0,0,0]}^{T}$.

As the selected point locates in the gap, a higher order expansion is used here, which is

$H_{e}=\delta_{IJ}E_{I}^{0}+\left\langle\left. I \right| \right.\frac{\partial H}{\partial\boldsymbol{k}}d\boldsymbol{k}\left| \left. J \right\rangle\right.-\sum_{K\neq I,J} \frac{\left\langle\left. I \right| \right.\frac{\partial H}{\partial\boldsymbol{k}}d\boldsymbol{k}\left| \left. K \right\rangle\right.\left\langle\left. K \right| \right.\frac{\partial H}{\partial\boldsymbol{k}}d\boldsymbol{k}\left| \left. J \right\rangle\right.}{E_{K}^{0}-1/2(E_{I}^{0}+E_{J}^{0})}$.

Then, we got the matrix form of the effective Hamiltonian

$H_{e}=\left[ \begin{matrix} \frac{4}{3} +5 {p_{x}}^{2}+10 p_{y}+\frac{227}{3}{p_{y}}^{2}+\frac{57}{2}{p_{z}}^{2} & p_{x} (\frac{-9}{5}-\frac{21}{4} p_{y}+i \frac{1}{2}p_{z}) \\ p_{x}(\frac{-9}{5}-\frac{21}{4} p_{y}-i \frac{1}{2}p_{z}) & \frac{2}{3} +\frac{5}{3} {p_{x}}^{2}+\frac{5}{4} p_{y}-\frac{57}{8}{p_{y}}^{2}+\frac{1}{2}{p_{z}}^{2} \end{matrix} \right]$,

which can also be written as

$H_{e}=\frac{48+160 {p_{x}}^{2}+270 p_{y}+1645{p_{y}}^{2}+696{p_{z}}^{2}}{48}\sigma_{0}-\frac{16+80 {p_{x}}^{2}+210 p_{y}+1987{p_{y}}^{2}+672{p_{z}}^{2}}{48}\sigma_{x}-p_{x}( \frac{9}{5}+\frac{21}{4} p_{y})\sigma_{y}-\frac{1}{2}p_{x}p_{z}\sigma_{z}$.

To show the nature of these band, the $\sigma_{0}$ and ${p_{x,y,z}}^{2}$ terms can be neglected. Then we get a simpler form as

$$H_{e}=-\frac{16+210 p_{y}}{48}\sigma_{x}-p_{x}( \frac{9}{5}+\frac{21}{4} p_{y})\sigma_{y}-\frac{1}{2}p_{x}p_{z}\sigma_{z}$$

This is the full form of the Eq. (2) in the main text.

V. Effective Hamiltonian of the nodal line system

By removing the TRS terms in the effective permittivity tensor, namely the $\epsilon_{d}$ and $\epsilon_{s}$ in the Eq.(7) of the main text, we get the nodal line system as exhibited in the Fig.2 of the main text. Here, we show the full Hamiltonian of this system can be expressed as

$H=\left[ \begin{matrix} 0 & 0 & 0 & 0 & k_{z} & -k_{y} & \omega_{p} & 0 & 0 & 0 \\ 0 & 0 & 0 & -k_{z} & 0 & k_{x} & 0 & \omega_{p} & 0 & 0 \\ 0 & 0 & 0 & k_{y} & {-k}_{x} & 0 & 0 & 0 & 0 & 0 \\ 0 & -k_{z} & k_{y} & 0 & 0 & 0 & 0 & 0 & 0 & 0 \\ k_{z} & 0 & -k_{x} & 0 & 0 & 0 & 0 & 0 & 0 & 0 \\ -k_{y} & k_{x} & 0 & 0 & 0 & 0 & 0 & 0 & 0 & 0 \\ \omega_{p} & 0 & 0 & 0 & 0 & 0 & 0 & 0 & \omega_{0} & 0 \\ 0 & \omega_{p} & 0 & 0 & 0 & 0 & 0 & 0 & 0 & \omega_{0} \\ 0 & 0 & 0 & 0 & 0 & 0 & \omega_{0} & 0 & 0 & 0 \\ 0 & 0 & 0 & 0 & 0 & 0 & 0 & \omega_{0} & 0 & 0 \end{matrix} \right]$.

For simplicity, $\epsilon_{0}=1$, $\mu_{0}=1$, and $\sqrt{\omega_{0}^{2}-\alpha\left( k_{x}^{2}+k_{y}^{2} \right)}\to\omega_{0}$ are applied in this matrix form as what used in the above derivation.

A representative case can be obtained by choosing $\omega_{p}=\omega_{0}=1$. Under this condition, we get the nodal line as $k_{x}^{2}+k_{y}^{2}=2$. Then, we choose $(k_{x},k_{y},k_{z})=(0,\sqrt{2},0)$, which is exactly on the nodal line, to examine its effective Hamiltonian. Here, the two degenerated modes are in form

$\left| \left. I \right\rangle\right.={[0,1,0,0,0,0,0,\sqrt{2},0,1]}^{T}$;

$\left| \left. J \right\rangle\right.={[0,0,1,1,0,0,0,0,0,0]}^{T}$.

Substituting $\left| \left. I \right\rangle\right.$ and $\left| \left. J \right\rangle\right.$ into the first order expansion equation (see the above section), the effective Hamiltonian is obtained as

$H_{e}=\left[ \begin{matrix} \sqrt{2} & -p_{z} \\ -p_{z} & \sqrt{2}+2 p_{y} \end{matrix} \right]$,

which can also be written as

$H_{e}=(\sqrt{2}+ p_{y})\sigma_{0}-p_{y} \sigma_{x}-p_{z} \sigma_{z}$.

This is the full form of the Eq.(1) in the main text.

**Fig. S1.** Field plots of the TE and LP modes of the Weyl metamaterial. Cross sectional views of these modes in the x-z plane are presented, where arrows show the distribution of electric field ($E_{x},E_{y}, E_{z}$). These results are obtained along the $\Gamma-X$ direction ($k_{x}\neq0$, $k_{y}=k_{z}=0$) at frequency 26 GHz (higher than the Weyl frequency).


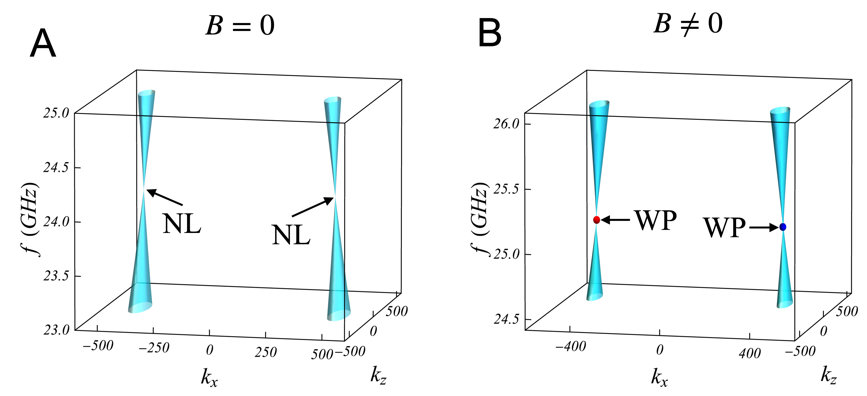


**Fig. S2.** 3D views of the band structures in the $k_{x}-k_{z}$ plane for the NL and Weyl metamaterials, respectively.


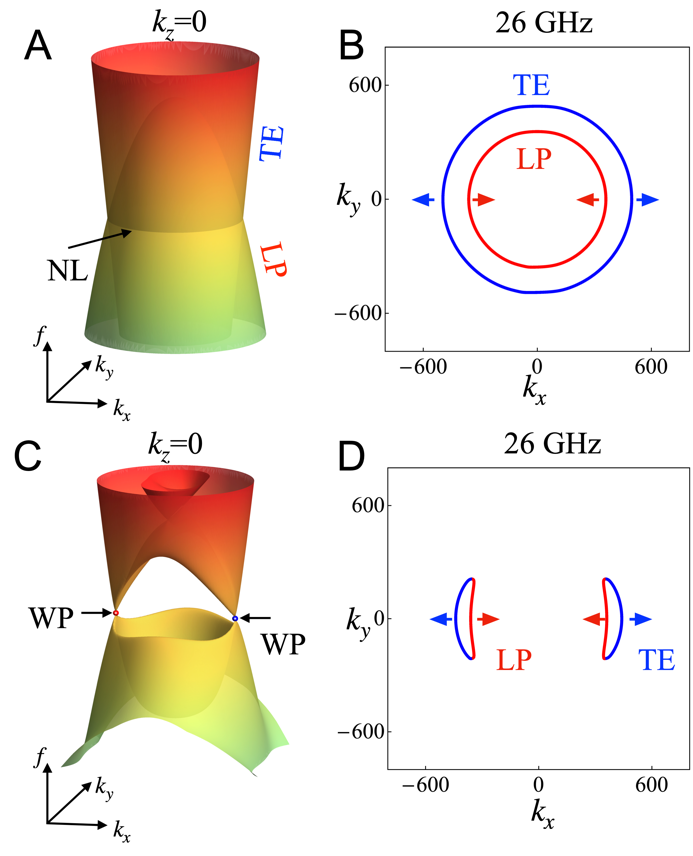


Fig. S3. A and C Dispersions of the NL system and Weyl system obtained by using the effective Hamiltonians respectively. The TE mode and LP mode are labeled in the NL system. B and D EFCs of the effective NL system and Weyl system calculated in the $\boldsymbol{k}_{\boldsymbol{x}}\boldsymbol{-}\boldsymbol{k}_{\boldsymbol{y}}$ plane at a fixed frequency (26 GHz), which is slightly above the NL (or Weyl) frequency. The TE mode and LP mode are marked in blue and red respectively. Colored small arrows show the directions of group velocity of the different modes.


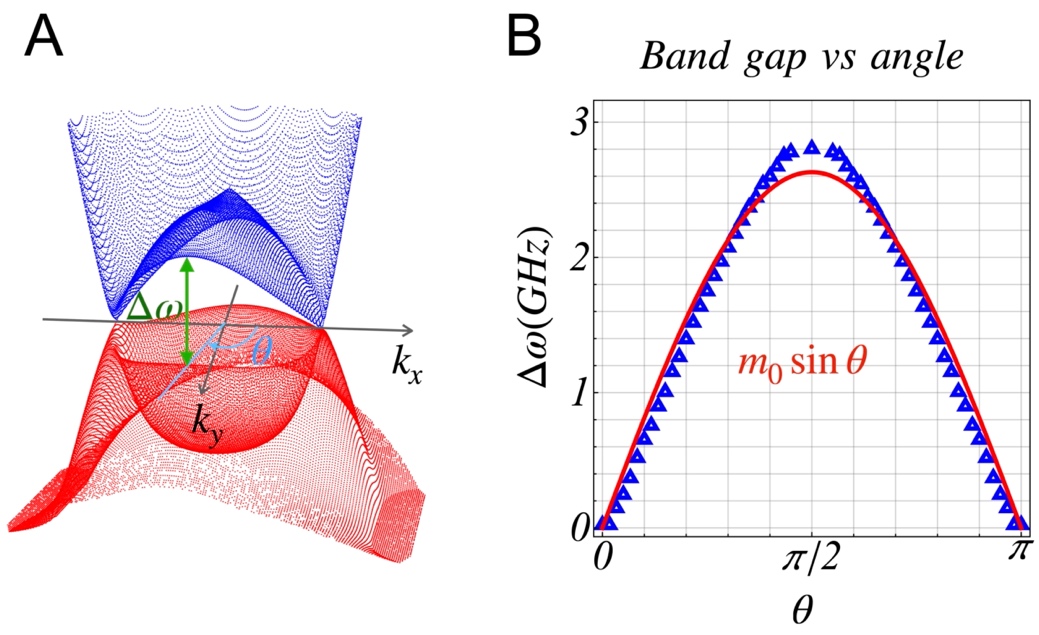


Fig. S4. The bandgap $\boldsymbol{\Delta}\boldsymbol{\omega}$ along the azimuth direction $\boldsymbol{\theta}$ is measured. A The schematic shows the bandgap $\boldsymbol{\Delta}\boldsymbol{\omega}$ and the azimuth angle $\boldsymbol{\theta}$. B The magnitude of bandgap can be fitted by a sinusoid with period $\mathbf{2}\boldsymbol{\pi}$.

Fig. S5. The influence of the nonlocal effect in our effective medium model. A non-zero factor of nonlocal effect ($\boldsymbol{\alpha}$) will make the variation of LP band from a flat line (LP1) to a downward curve (LP2), which leads to the change of the Weyl cone (band crossing). A type-I Weyl point is presented only when the nonlocal effect is considered.

**
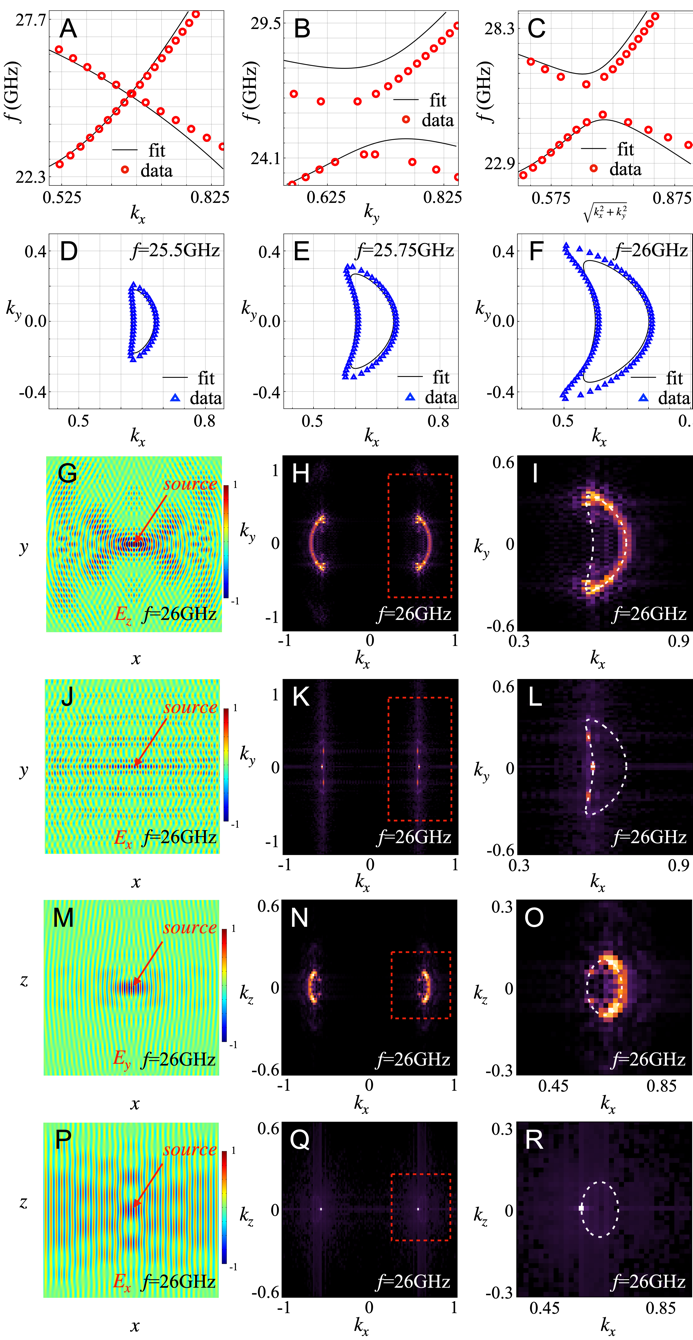
Fig. S6.** Comparison between the results of real structure and effective medium (EM). **A**-**C** are band structures along the $k_{y}=k_{z}=0$, $k_{x}=k_{z}=0$, and $k_{x}-k_{y}=k_{z}=0$ directions, respectively. Results for real structure and EM are indicated by the red circles and black lines correspondingly. **D**-**F** are EFCs calculated from the real structure (blue triangles) and EM model (black lines) on frequencies $25.5 GHz$, $25.75 GHz$, and $26 GHz$, respectively. **G** Simulated bulk electric field [$Re(E_{z})$] in the $x-y$ plane based on fitted EM parameters. A point source of waves (electric dipole in $z$ direction) is located at the center of plane, and the region of simulation is a square with width $50 \lambda_{0}$ ($\lambda_{0}$, the vacuum wavelength). The operating frequency is $f=26 GHz$. **H** The Fourier transformed result of **G**. **I** Zoom-in image of the red frame in **H**. White dashed line is the calculated EFC on $f=26 GHz$ of the fitted Weyl EM. **J**-**L** are the corresponding results of **G**-**I** obtained by plotting the $Re(E_{x})$ field. **M**-**O** are the field [$Re(E_{y})$] and Fourier transformed results on the section of $x-z$ plane with a wave source (electric dipole) in $y$ direction. **P**-**R** are the corresponding results of **M**-**O** obtained by plotting the $Re(E_{x})$ field. $k_{x}$ and $k_{y}$ are scaled with $k_{0}=\frac{2\pi}{5\times{10}^{-3}}$ in these results.


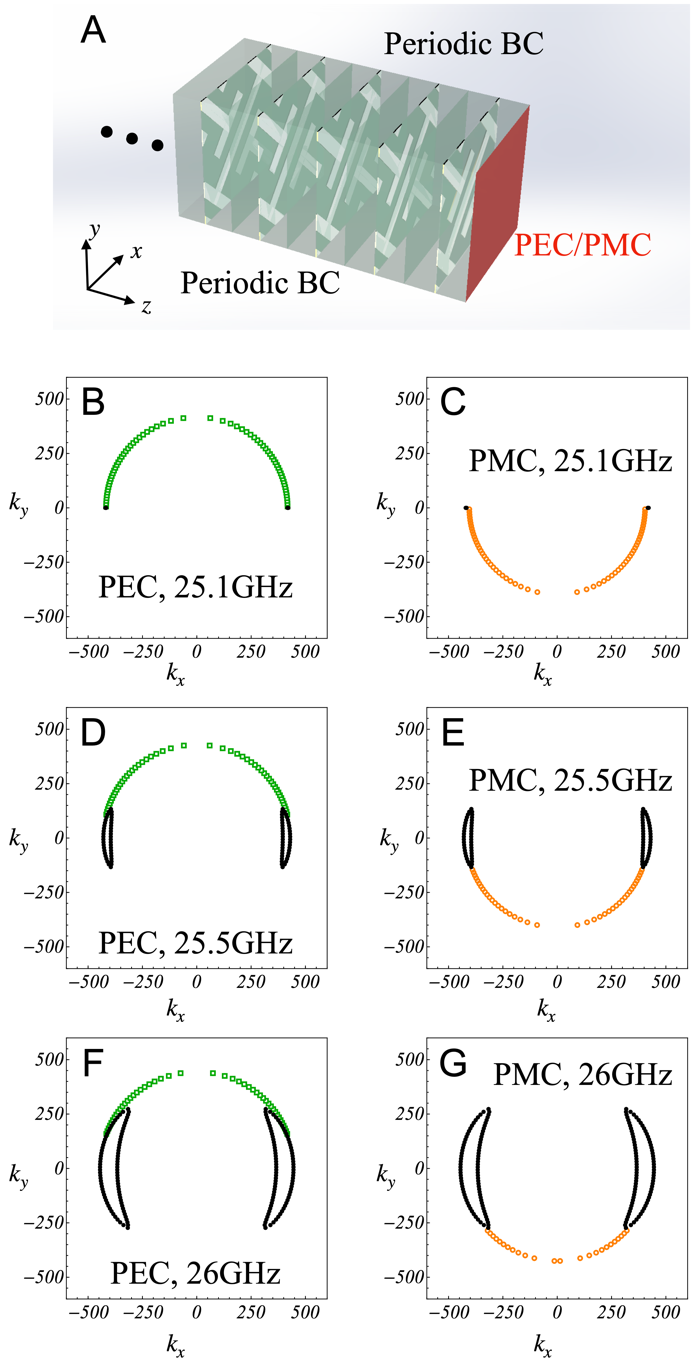


Fig. S7. Simulated surface Fermi arcs on the PEC and PMC boundaries of the real metallic structure. A The unit cell and related boundary conditions (BC) used for this simulation. B and C The simulated surface Fermi arcs at frequency 25.1 GHz. D and E The simulated surface Fermi arcs at frequency 25.5 GHz. F and G The simulated surface Fermi arcs at frequency 26 GHz. Green squares (orange circles) show the simulated surface states on PEC (PMC) boundary, black dots are simulated bulk states on the corresponding frequency.


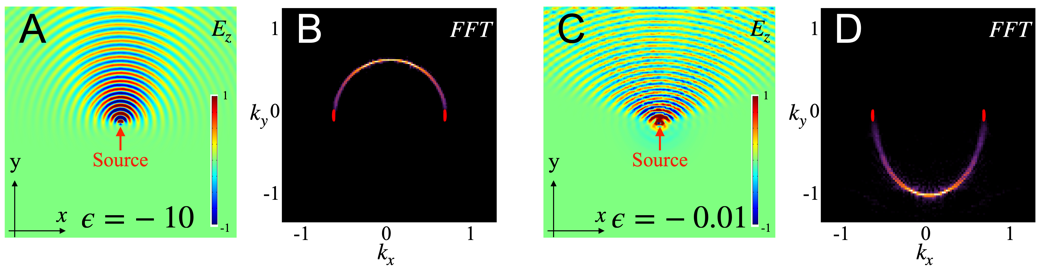


Fig. S8. Distributions of electric field and FFT results obtained on the interfaces between the Weyl media and dielectric materials with permittivities $\boldsymbol{\epsilon=-10}$ and $\boldsymbol{-0.01}$ at frequency 25 GHz. These interfaces mimic the PEC and PMC boundary conditions with realistic parameter respectively. $\boldsymbol{k}_{\boldsymbol{x}}$ and $\boldsymbol{k}_{\boldsymbol{y}}$ are scaled with $\boldsymbol{k}_{\boldsymbol{0}}\boldsymbol{=}\frac{\boldsymbol{2\pi}}{\boldsymbol{5\times}\boldsymbol{10}^{\boldsymbol{-3}}}$.


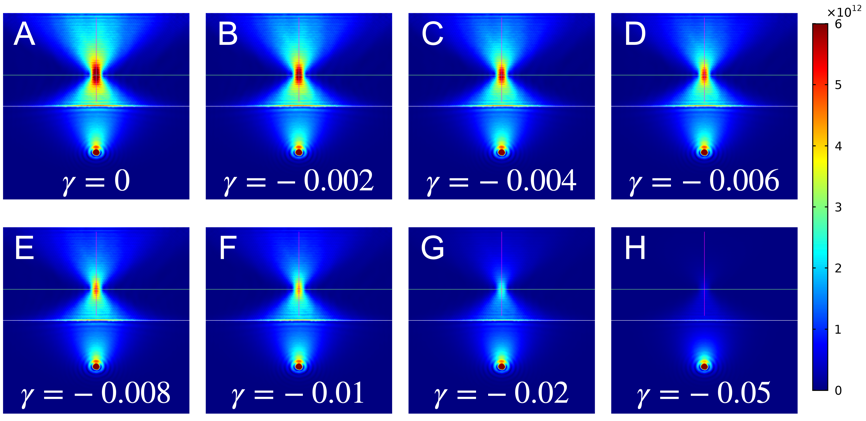


Fig. S9. All-angle reflectionless negative reflection observed under different material loss $\boldsymbol{\gamma}$. Magnitudes of the electric field $\left| \boldsymbol{E} \right|$ are plotted here. PEC and PMC boundary conditions are applied for the lower and upper surfaces respectively. An electric dipole (25 GHz) is placed at the lower part to serve as the source of surface wave. Green and magenta lines in the upper part show the positions of field which are plotted in Fig. S11.


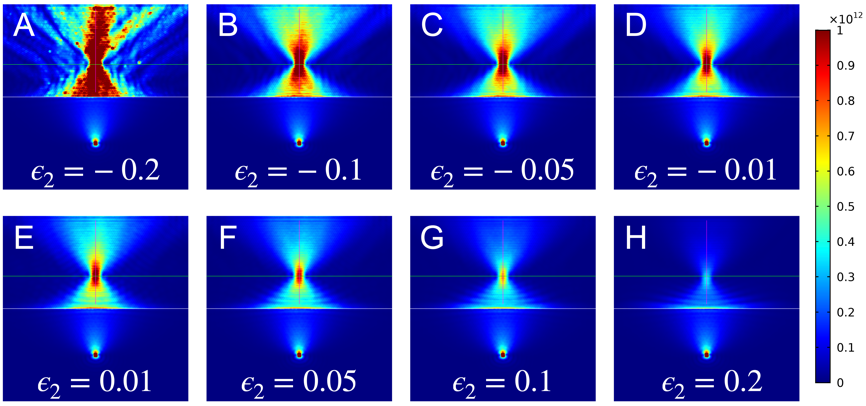


Fig. S10. Reflectionless negative reflection observed for different values of permittivity $\boldsymbol{\epsilon}_{\boldsymbol{2}}$ which approximates the PMC boundary condition. Magnitudes of the electric field $\left| \boldsymbol{E} \right|$ are plotted here. Dielectric layers with $\boldsymbol{\epsilon}_{\boldsymbol{1}}\boldsymbol{=-10}$ and $\boldsymbol{\epsilon}_{\boldsymbol{2}}$ are applied for the lower and upper surfaces respectively. An electric dipole (25 GHz) is placed at the lower part to serve as the source of surface wave. Green and magenta lines in the upper part show the positions of field which are plotted in Fig. S11.


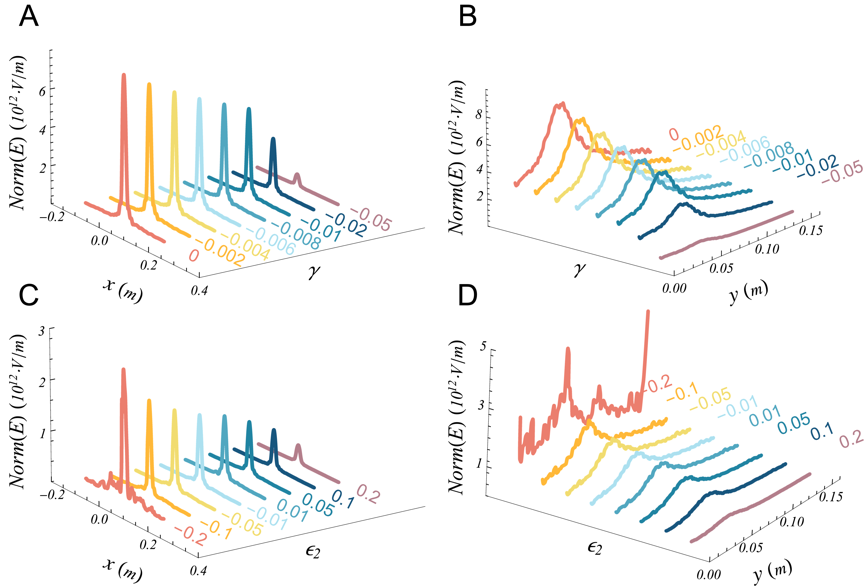


Fig. S11. A and B The transverse profiles of electric field $\left| \boldsymbol{E} \right|$ on the focus points along $x$ and $y$ directions respectively. Different values of material loss $\gamma$ are used in these simulations where the PEC and PMC boundary conditions are applied. C and D Dielectric layers with $\epsilon_{1}=-10$ and $\epsilon_{2}$ are used to approximate the PEC and PMC boundaries correspondingly where different values of $\epsilon_{2}$ are examined in calculating these transverse profiles. See also Figs.S9 and S10 for details.
